# Clinical experience with percutaneous tibial nerve stimulation in the elderly; do outcomes differ by gender?

**DOI:** 10.1080/2090598X.2019.1590032

**Published:** 2019-04-08

**Authors:** Cristina Palmer, Nobel Nguyen, Gamal Ghoniem

**Affiliations:** Division of Female Urology and Voiding Dysfunction, Department of Urology, University of California Irvine, Orange, CA, USA

**Keywords:** Female urology, overactive bladder, voiding dysfunction, neuromodulation, PTNS, elderly/geriatrics

## Abstract

**Objective**: To evaluate the use of percutaneous tibial nerve stimulation (PTNS) in an elderly population, as PTNS is a third-line treatment in the management of overactive bladder (OAB) and affects 10–26% of adult males and 8–42% of adult females, increasing in prevalence with age.

**Patients and methods**: We performed a chart review of patients aged ≥ 65 years undergoing PTNS at a single institution over 6 years. We examined clinicopathological variables potentially associated with the outcomes of interest.

**Results**: In total, 52 patients aged ≥ 65 years underwent an induction course of PTNS between 2011 and 2017, comprising 23 men and 29 women. The mean age of the patients was 75.75 years and the mean body mass index (BMI) was 26.33 kg/m^2^. In all, 36 patients used anticholinergic treatments prior to PTNS, five used a β_3_-adrenoceptor agonist, and three had Botox injections. After PTNS, 37 patients reported improvement of their symptoms, with 21 using combined therapy during PTNS. Only seven patients used an anticholinergic after PTNS, six used a β_3_-adrenoceptor agonist, five had Botox injections, and two had sacral neuromodulation. When looking at variables such as age, gender, race, BMI, and comorbidities, we found that an obese BMI was the only statistically significant variable predicting failure of response. A sub-analysis of only women did not demonstrate any predictors of failure.

**Conclusion**: Our subjective response rate of 70% was within the success rates reported in literature. In all, 39% of patients used a concomitant treatment during PTNS and 13.2% required alternative treatment after PTNS.

**Abbreviations**: BMI: body mass index; OAB: overactive bladder; PTNS: percutaneous tibial nerve stimulation; UI: urinary incontinence

## Introduction

Overactive bladder (OAB) syndrome is defined by the ICS as ‘urinary urgency, usually accompanied by frequency and nocturia, with or without urgency urinary incontinence, in the absence of urinary tract infection (UTI) or other obvious pathology’ [1]. It is a condition that has been estimated to affect up to 34 million Americans, with estimates of 546 million people affected worldwide by 2018, and can have significant detrimental effects on quality of life [2]. It is also known that the prevalence of OAB increases with age [3,4]. The prevalence of urinary incontinence (UI) in women has been shown to increase through adulthood, with up to 39% of women aged > 60 years with daily urge UI [4]. Men have a lower prevalence, with up to 11% for daily urge UI episodes when aged > 65 years [4].

Recommended first-line treatment for OAB includes behavioural therapy, which should be offered to all patients. Behavioural modifications may then be combined with pharmacological therapy in an attempt to minimise symptoms and improve quality of life; however, there is a high discontinuation rate of anticholinergics, with reports of only 18% of patients compliant after 6 months [5,6]. Third-line treatment options include intravesical onabotulinumtoxinA injections, sacral neuromodulation, or percutaneous tibial nerve stimulation (PTNS) [5]. PTNS was approved for treatment of OAB in 2000. It acts by stimulating the afferent fibres of the tibial nerve (L4–S3). It is a less invasive form of neuromodulation therapy than sacral neuromodulation; however, it requires a motivated patient to return weekly for 12 weeks [6].

The elderly population is unique, with increased medical comorbidities and the possibility for cognitive and functional deficits. To date, there is a paucity of studies looking at this specific patient population regarding the PTNS treatment modality. Our primary aim in the present study was to describe our clinical experience with PTNS in an elderly population and report treatment response, concomitant therapies, and alternative treatments needed after therapy. Our secondary aim was to look specifically at elderly women, to determine if there is a difference in response based on gender.

## Patients and methods

This is a retrospective chart review of patients aged ≥ 65 years undergoing a 12-session induction course of PTNS at a single institution over a 6-year period between 2011 and 2017. The study was approved by the Institutional Review Board of the University of California, Irvine (HS#2016–2976). Using the Current Procedural Terminology (CPT) codes for PTNS (64,566), a procedure total of 499 charts were identified and reviewed. Only patients undergoing PTNS for idiopathic OAB, and who had completed the 12-week session treatment were included. We excluded neurogenic bladder, urinary retention, or patients with chronic pelvic pain syndrome. A total of 52 patients were included in the analysis who met the inclusion criteria. Demographic data included patient’s age, sex, body mass index (BMI), race, and medical comorbidities. Assessment of the patients’ OAB symptoms were obtained at baseline and after the induction course of PTNS using the validated eight-item OAB questionnaire (OAB-V8), 3-day voiding diary, as well as the Patient Global Impression of Satisfaction (PGI-S) after treatment. In addition, we recorded if patients required concomitant therapy during PTNS, as well as if alternative therapy, such as intravesical onabotulinumtoxinA injections, anticholinergic or β-agonist medications, or sacral neuromodulation was needed following PTNS treatment.

**Figure 1.**
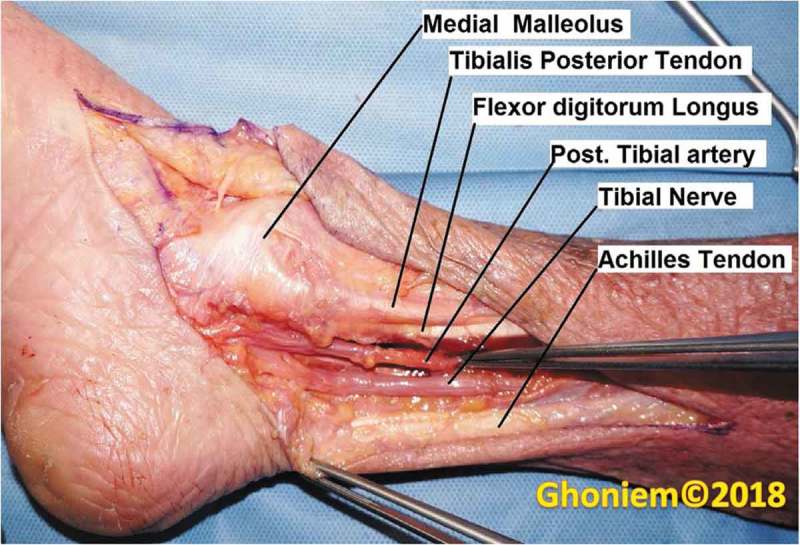
Anatomy of the tibial nerve in relation to the medial malleolus.

Briefly, the tibial nerve is located just anterior to the Achilles tendon cephalad to the medial malleolus [7]. PTNS is performed by placing a 34-G needle 3–5 cm cephalad to the medial malleolus and posterior to the tibia, at 60 ° (Figure 1) [8,9]. A grounding pad is placed on the bottom of the foot, after which a low-voltage stimulator is attached to the needle. Correct needle placement is confirmed by observing dorsiflexion of the big toe or a fanning of the toes, as well as patient-reported sensation on the bottom of the foot. Stimulation is typically performed at 0.5–9.0 mA amplitude at a fixed frequency of 20 Hz and a set pulse width of 200 µs, based on patient sensation, motor response, and comfort [6,9]. The induction treatment course consists of 12 consecutive weekly sessions, for 30 min per session. If patients report a good response, measured as a > 50% improvement in symptoms, they may continue with once monthly maintenance treatment.

Statistical analysis was performed using an unpaired *t*-test for continuous variables, summarised with mean, SDs, and SEMs. Categorical variables, such as medical comorbidities, gender, and race, were analysed using chi-squared analysis. A logistic regression, multivariate analysis was performed to identify predictive factors for patients who would benefit most from PTNS treatment. *P* values generated from chi-squared tests for categorical variables and *t*-tests for continuous variables were reported. A *P* < 0.05 was considered to indicate statistical significance.

## Results

A total of 499 charts were reviewed. After accounting for multiple visits per induction of treatment course, 52 patients were eligible for inclusion. In all, 23 of the patients were men (44.3%) and 29 were women (55.8%). The mean (range) age of patients was 75.75 (65–93) years and BMI was 26.33 (17.4–43.9) kg/m^2^. In all, 34 patients self-identified as White, and 18 identified as non-White. There were 18 patients (34%) with one to two medical comorbidities, 22 (43.4%) with three to four medical comorbidities, and 12 (22.6%) had more than five comorbidities. Anticholinergic treatments were used by 36 patients (69%) prior to PTNS, five used a β_3_-adrenoceptor agonist, and three had intravesical onabotulinumtoxinA injections. Overall, 21 patients (39%) used combined therapy during PTNS. After PTNS, 37 patients (70%) reported improvement of their symptoms. Only seven patients used an anticholinergic after PTNS, six patients used a β_3_-adrenoceptor agonist, five had intravesical onabotulinumtoxinA injections, and two underwent sacral neuromodulation.

Of the patients who reported improvement with PTNS, the average BMI was 25.08 kg/m^2^. The average BMI of patients without improvement with PTNS was 29.23 kg/m^2^. When looking at variables such as age, gender, race, BMI, and comorbidities, we found an obese BMI (≥ 30 kg/m^2^) to be the only statistically significant variable predicting failure of response (*P* = 0.002). We then performed a logistic regression, multivariate analysis and found that none of these factors were predictive of the improvement of treatment after PTNS. Of elderly patients who received intravesical onabotulinumtoxinA injections with continued symptoms, all reported symptom improvement following PTNS.

We then performed a sub-analysis on women in the cohort. With women aged > 65 years, the average age was 74.2 years, with an average BMI of 28.2 kg/m^2^. In our group of elderly women, 13 had one to two medical comorbidities, 10 had three to four medical comorbidities, and six had more than five comorbidities. In all, 20 women (69%) used anticholinergic treatments prior to PTNS, one used a β_3_-adrenoceptor agonist, and one had intravesical onabotulinumtoxinA injections. There were 11 women (38%) who used combined therapy during PTNS. After PTNS, 19 women (66%) reported improvement of their symptoms. Only two women (7%) used an anticholinergic after PTNS, six used a β_3_-adrenoceptor agonist, four had intravesical onabotulinumtoxinA injections, and none of the women underwent sacral neuromodulation. These values are similar to the overall cohort with both genders combined. However, when looking at women alone, BMI was not a statistically significant variable predicting failure of response (*P* = 0.883).

## Discussion

In our present population of elderly patients undergoing PTNS for OAB, we found a subjective success rate of 70% overall and 66% for women only, which is well within the established success rates reported in the literature of 37–82% [6]. Our present study demonstrated a significant decrease in the use of anticholinergics following PTNS, as 36 (67.9%) patients used an anticholinergic prior to therapy and only seven (13.2%) used an anticholinergic after therapy. An obese BMI was the only variable predictive of failure in our present cohort, which was the same in our control group. This was not predictive of failure in women alone. An interesting finding was that all of the patients who had failed intravesical onabotulinumtoxinA injections had a favourable response after completing the PTNS course. In elderly women only, the results were similar, demonstrating good success in both men and women.

Our present study compares to a meta-analysis of four non-comparative studies with a pooled subjective success rate of 60.6% [6]. It also has similar findings to those of George et al. [10], who reported that an obese BMI trended towards being a predictive factor for patients undergoing sacral neuromodulation benefiting from supplemental treatment with an anticholinergic. They hypothesise that this may be due to increased difficulty when placing the electrode in an ideal location [10]. In contrast, Peters et al. [11] found that those undergoing re-operation for sacral neuromodulation had a statistically lower BMI. We hypothesise that a higher BMI may predict a lower success rate in our patients undergoing PTNS due to the increased amount of tissue that the voltage conducts through. Amundsen et al. [12] found that patients with three or more medical comorbidities had a lower cure rate after sacral neuromodulation; however, our present study did not show an association between number of comorbidities and response, in either the combined gender cohort or women alone.

The present investigation is important as the population continues to age. According to the USA Census Bureau, 2012 National Projections predict significant population growth between 2012 and 2050, with 83.7 million people becoming aged ≥ 65 years [13]. This value is almost double the elderly population of 43.1 million in 2012 [13]. This will have a significant impact on healthcare and predicted need for adjustments to deliver optimal patient-centric care. Anticholinergics have been a mainstay for the treatment of OAB [14,15]. However, compliance is low due to potential side-effects [15,16]. In 1991, the American Geriatrics Society first published the Beers Criteria for Potentially Inappropriate Medication Use in Older Adults, with the most recent update published in 2015 [17]. Anticholinergics are included in this evidence-based list of medications that should be avoided in older adults, as this class of drugs also has an increased risk of cognitive decline, with the strength of the recommendation rated as ‘strong’ [17]. Clinicians should also avoid prescribing this class of medication in older adults to avoid the risk of polypharmacy [18]. Our present study demonstrates that PTNS is a viable, minimally invasive option for the treatment of OAB in an elderly population, to minimise the addition of another medication in a population who may be prone to increased adverse effects due to medications.

We recognise some limitations to our present study. This is a retrospective descriptive study and has a small sample size, which perhaps weakens our study’s statistical power and results. The present study could be strengthened by using objective measures of success to PTNS, such as changes in bladder diary or urodynamic parameters. The use of additional validated questionnaires would have strengthened the analysis as well. Finally, data were collected retrospectively and thus were subject to the flaws inherent in a retrospective design, such as recall or misclassification bias, as well as subject to other confounding variables. However, all laboratory and clinical data for the patients were recorded in the electronic ambulatory medical charts. The collection of data by researchers was strict and thorough to minimise the bias and flaws of retrospective studies. PTNS therapy requires a motivated patient with the time for weekly follow-up for 12 weeks, as well as a reliable means of transportation. We recognise this is not always feasible for an elderly population.

## Conclusion

In conclusion, the present study highlights the effectiveness and viability of PTNS as a treatment alternative for OAB in the elderly population. Special consideration should be taken for this unique patient population. We suggest that current treatment algorithms for OAB be modified for the elderly, as PTNS may be an appropriate second-line treatment option, over pharmacotherapy. Although, we believe that treatment selection should be a patient-centric decision, it is not necessarily to be in a step-wise manner. Beside that, if the patient switches to another line it is not necessary to stop the treatment of the other line for example; we still encouraged patients to continue the behavioural modification and do PTNS and/or other second- or third-line OAB treatments. Our experience with PTNS in this population demonstrates a large decrease in anticholinergic use following PTNS, in conjunction with high subjective success rates. In addition, PTNS should be kept as an option for those who fail intravesical onabotulinumtoxinA injections.
