# Correction

**DOI:** 10.1080/2090598X.2019.1620500

**Published:** 2019-05-30

**Article title**: Clinical outcomes of percutaneous tibial nerve stimulation in elderly patients with overactive bladder

**Authors**: Palmer, C., Farhan, B., Ngyuen, N., & Ghoniem, G.

**Journal**: *Arab Journal of Urology*

**Bibliometrics**: Volume 17, Number 1, pages 10–13

**DOI**: https://doi.org/10.1080/2090598X.2019.1590032

When this article was first published, it contained the following errors:

- The title had been incorrectly presented as follows: ‘Clinical experience with percutaneous tibial nerve stimulation in the elderly; do outcomes differ by gender?’
- The second author, Bilal Farhan, was missing from the author list.
- Cristina Palmer was incorrectly listed as the corresponding author instead of Gamal Ghoniem.

The article has now been corrected as follows:

- The title has been corrected to the following: ‘Clinical outcomes of percutaneous tibial nerve stimulation in elderly patients with overactive bladder’.
- Bilal Farhan has been added to the author list and the Disclosure statement has also been amended to include this author’s details.
- The corresponding author has been changed to Gamal Ghoniem.

